# Compact Integration of a GSM-19 Magnetic Sensor with High-Precision Positioning using VRS GNSS Technology

**DOI:** 10.3390/s90402944

**Published:** 2009-04-22

**Authors:** Angel Martín, Jorge Padín, Ana Belén Anquela, Juán Sánchez, Santiago Belda

**Affiliations:** Department of Cartographical Engineering, Geodesy and Photogrammetry. Polytechnic University of Valencia. Camino de Vera s/n, 46022, Valencia, Spain; E-Mails: jpadin@cgf.upv.es; anquela@cgf.upv.es; juansans3@topo.upv.es; sanbelp1@topo.upv.es

**Keywords:** Magnetic data, GNSS positioning, VRS approach, sensor integration

## Abstract

Magnetic data consists of a sequence of collected points with spatial coordinates and magnetic information. The spatial location of these points needs to be as exact as possible in order to develop a precise interpretation of magnetic anomalies. GPS is a valuable tool for accomplishing this objective, especially if the RTK approach is used. In this paper the VRS (Virtual Reference Station) technique is introduced as a new approach for real-time positioning of magnetic sensors. The main advantages of the VRS approach are, firstly, that only a single GPS receiver is needed (no base station is necessary), reducing field work and equipment costs. Secondly, VRS can operate at distances separated 50–70 km from the reference stations without degrading accuracy. A compact integration of a GSM-19 magnetometer sensor with a geodetic GPS antenna is presented; this integration does not diminish the operational flexibility of the original magnetometer and can work with the VRS approach. The coupled devices were tested in marshlands around Gandia, a city located approximately 100 km South of Valencia (Spain), thought to be the site of a Roman cemetery. The results obtained show adequate geometry and high-precision positioning for the structures to be studied (a comparison with the original low precision GPS of the magnetometer is presented). Finally, the results of the magnetic survey are of great interest for archaeological purposes.

## 1. Introduction

Magnetometry is one of the most common tools used in geophysical research for prospecting and archaeology due to the rapidity of data collection and the general applicability of the technique to shallow research [1].

Different kinds of magnetometers can be found [1–3]; fluxgate magnetometers can be used to measure any component of the geomagnetic field and currently, depending on the sensor alignment, most of them achieve a sensitivity of 0.1 nT and can measure the field continuously. Proton-precession magnetometers measure the Earth’s total field; their advantage over fluxgate magnetometers is that sensor alignment is not critical. However, the measurements with this tool cannot be continuous, as the proton-precession magnetometer requires several seconds due to the polarizing and relaxing time needed by the protons, and currently most of them achieve a sensitivity of 0.1 nT. Proton-precession magnetometers with Overhauser-effect (such as the GSM-19 instrument used in this work) can achieve two measurements per second with 0.2 nT absolute accuracy over its full temperature range. Alkali-vapor magnetometers, usually used for airborne gradiometers, measure the Earth’s total field. The orientation of this instrument is not critical as they have a high sensitivity, on the order of 0.01 nT.

The majority of magnetometer surveys require high-density data and accurate positioning. The most common procedure is to divide the survey area (which is usually small) into data grids. This procedure requires the physical placement of these grids in the field, which increases the work time.

If precise data location could be collected simultaneously with magnetometer data collection, the additional time required for grid implementation could be avoided because no grid materializations should be needed. The most appropriate tool to carry that out is GPS. The use of GPS for navigation and location is a common theme in many prospecting applications, for example as described in [5].

Currently, it is easy to find magnetometers with GPS of different precisions and prices [4]. Stand alone options can obtain accuracies of approximately five meters and stand alone can combine WAAS/EGNOS augmentations, achieving precisions of better than one meter, [6]. The best precision (better than 0.1 m) can be obtained with a DGPS-RTK system, but this system requires another GPS device working in the area, and this implies both an increase in time needed and a significant larger budget. Recently, real-time kinematic (RTK) technology has been used in magnetic surveys, [7,8]. Following this procedure, the integration of magnetic data with GPS using a VRS (Virtual Reference Station) instead of RTK technology is presented. VRS technology requires less equipment, time dedication and information than RTK, because differential corrections are sent by the GNSS reference network control centre using the Internet. That means that no master station located on known coordinates is needed near the prospection area. In terms of accuracy, RTK and VRS offer the same results [9].

## 2. VRS concept

The VRS concept, [10–13] is a derivative of the multiple reference station approach, [14,15]. The main difference is that the surrounding reference GPS stations are used to determine “synthetic” dual-frequency code and carrier-phase GPS at a virtual base station that is located close to the user’s receiver. These GPS reference stations are strategically located in an extensive geographical area and are connected to a network control centre. This centre receives information from the GPS stations in real-time and, using an NTRIP protocol, creates the differential corrections to be applied anywhere inside the geographical area where the GPS network is located.

A user connected to this network will receive the differential corrections and a virtual reference station is created near the rover position. This virtual reference station acts as the master station in RTK positioning.

The main benefit of the VRS approach is that it does not require a physical reference station close to the user (usually with known coordinates in the reference frame in which the survey will take place). However, it does require a reference GPS station infrastructure and a control centre.

The VRS approach is also more flexible in terms of permitting users to use their current receivers and software without involving any special software or communications equipment (if used in post-processed mode) and the number of instruments needed for field operations is greatly reduced.

Finally, with VRS, users within the network can operate at greater distances than with the conventional RTK GPS model without degrading accuracy. Under ideal conditions, the VRS approach can deliver single-point coordinate accuracies of a 3–5 centimetres for a rover separated 50–70 km from the reference stations.

## 3. Compact magnetometer and GPS integration

**Figure 1.**
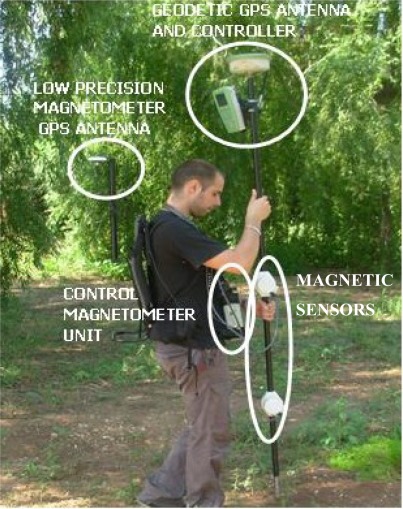
Magnetometer and geodetic GPS integration.

Figure 1 shows the compact integration of the GSM-19 magnetometer and the geodetic high precision GPS antenna. Only a screw to connect the staff with the antenna is needed.

No magnetic signal distortion is observed in any of the observations carried out with these coupled devices. That is, measurement time and signal amplitude numbers were under optimal conditions in the observation data files.

GSM-19 is equipped with a low precision GPS antenna. The static and kinematic precision of this antenna can be seen in Table 1, where static observations on control points (absolute error) and kinematic comparison with VRS results are presented. These control points can be seen in Figure 3.

As can be seen in Table 1, the magnetometer GPS does not guarantee high levels of accuracy, the quadratic component of absolute error introduces an error of 1.84 meters and the quadratic component of kinematic error of 0.86 meters (these are the expected results for this kind of GPS device). The data collected by the magnetometer is taken in Universal Coordinated Time (UTC) if the GPS option is activated on the control unit of the magnetometer. In this respect, correlation with the precise coordinates obtained with the geodetic antenna and VRS approach is made easier in post-process (the magnetic data and precise GPS coordinates share the same reference time system).

## 4. Field test

**Figure 2.**
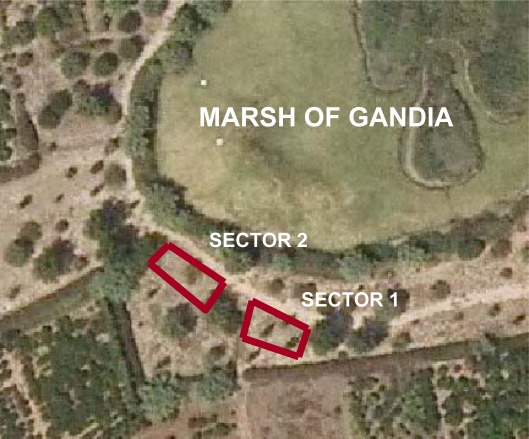
Survey area with the two prospected sectors. Image from Cartographical Institute of Valencia, image centre coordinates are 38°59′27″ N, 0°10′45″ W.

A magnetic survey was carried out in the Gandia marsh, near Gandia, a city located about 100 km south of Valencia (Spain). A Roman cemetery is thought to be buried in the survey area. The area is divided in two sectors due to grove layout. The sectors are small, approximately 10 by 5 meters (Figure 2).

One second was the time interval selected for data collection at 0.1–0.2 m/s walk speed, so the sample interval was about 0.25 m. The Active Geodetic GPS/GNSS network for the Valencia region [16,17] was used for VRS kinematic positioning. The data were collected walking in the same direction for every sector, so the orientation of the antenna is always the same.

Magnetic data were reduced for diurnal correction, magnetic anomalies were calculated by subtracting the mean magnetic value from the data and reduction to the pole was achieved. Kriging interpolation was used in order to display the magnetic anomalies in a 0.25 m × 0.25 m grid.

**Figure 3.**
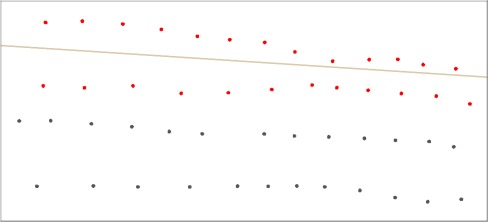
Control points located in the first sector. Brown line corresponds to the path. Black points are high precision GPS VRS antenna and red points are low precision magnetometer GPS antenna. The dimensions are 2.75 x 1.25 meters.

Comparison of coordinate accuracy can be seen in Figure 3. Some control points located in sector 1 have been observed with the high precision GPS VRS antenna (black points) and with the low precision GPS magnetometer antenna (red points). Taking the path through the area of reference, the low accuracy survey presents data points inside the path, but in the survey the limit of the path was never crossed. Based on this example, it can be concluded that the limits and forms of the magnetic anomalies can vary substantially depending on the precision of the coordinates of the observed points, which can lead to mistakes in their interpretation.

**Figure 4.**
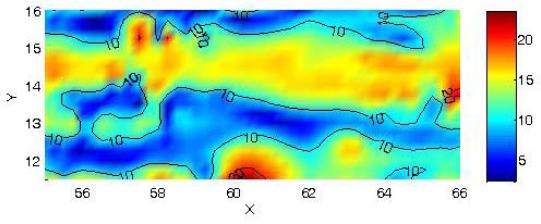
Magnetic anomalies in the first sector with precise geometry. Units in nT.

**Figure 5.**
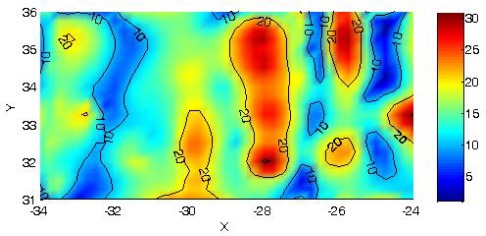
Magnetic anomalies in the second sector with precise geometry. Units in nT.

Figures 4 and 5 are the final plot of the magnetic survey for the first and second sector with high position accuracy. Original UTM coordinates have been translated and rotated only for a better plot representation.

In the first sector a marked magnetic anomaly is presented in the form of 11 by 1 meter corridor crossing the area horizontally. In the second sector some corridors can be seen crossing the plot vertically; these corridors have a mean width of approximately 1 meter.

## 5. Conclusions

The VRS GNSS technology is introduced as a very interesting approach not only for collection of magnetic data, but also for high-precision real-time data acquisition for positioning that can be extended to any geophysical research. It presents great advantages in terms of operational flexibility and cost-effectiveness. VRS requires only a single GPS receiver because no base station is necessary, reducing labor and equipment costs. It simplifies the operational logistics of field work and can operate at distances separated 50–70 kms from the reference stations without degrading accuracy. The results obtained in the magnetic survey with the compact integration of GSM-19 magnetic sensor with VRS GNSS technology are of great interest for archaeological purposes.

## Figures and Tables

**Table 1.** Absolute and kinematic error of the GSM-19 GPS antenna instrument.

	**Absolute error (m)**		**Kinematic error (m)**	
	**X**	**Y**	**X**	**Y**
**Mean**	1.110	1.467	−0.528	−0.317
**σ**	1.070	1.505	0.570	0.645
